# Antinociceptive Effect of Intrathecal Microencapsulated Human Pheochromocytoma Cell in a Rat Model of Bone Cancer Pain

**DOI:** 10.3390/ijms150712135

**Published:** 2014-07-08

**Authors:** Xiao Li, Guoqi Li, Shaoling Wu, Baiyu Zhang, Qing Wan, Ding Yu, Ruijun Zhou, Chao Ma

**Affiliations:** 1 Department of Rehabilitation Medicine, Sun Yat-sen Memorial Hospital, Sun Yat-sen University, Guangzhou 510120, China; E-Mails: lixiao204011987@sina.com (X.L.); 13660183777@126.com (S.W.); baiyuzsu@yahoo.com.cn (B.Z.); soswq@hotmail.com (Q.W.); 13654456833@163.com (D.Y.); zhouruijun6666@163.com (R.Z.); 2 Department of Cardiology, Sun Yat-sen Memorial Hospital, Sun Yat-sen University, Guangzhou 510120, China; E-Mail: liguoqi09@163.com

**Keywords:** human pheochromocytoma cell, cell transplantation, microencapsulation, analgesia, cancer-induced bone pain

## Abstract

Human pheochromocytoma cells, which are demonstrated to contain and release met-enkephalin and norepinephrine, may be a promising resource for cell therapy in cancer-induced intractable pain. Intrathecal injection of alginate-poly (l) lysine-alginate (APA) microencapsulated human pheochromocytoma cells leads to antinociceptive effect in a rat model of bone cancer pain, and this effect was blocked by opioid antagonist naloxone and alpha 2-adrenergic antagonist rauwolscine. Neurochemical changes of cerebrospinal fluid are in accordance with the analgesic responses. Taken together, these data support that human pheochromocytoma cell implant-induced antinociception was mediated by met-enkephalin and norepinephrine secreted from the cell implants and acting at spinal receptors. Spinal implantation of microencapsulated human pheochromocytoma cells may provide an alternative approach for the therapy of chronic intractable pain.

## 1. Introduction

Cancer-induced bone pain (CIP) is the most common cause of cancer pain [1,2], and approximately 70% of patients with terminal breast or prostate cancer have evidence of bone metastases [3]. Considering that terminal cancers considerably shorten the life expectancy of patients, relieve of pain is a preferential strategy for improving life quality. However, systemic opioid therapy fails to produce adequate analgesia and influence the prevalence of pain in CIP patients because of persistence of non-reversible undesirable side effects [4,5,6].

During the past three decades, cell therapy as a novel approach to treat pain has been tested in clinical pain treatment [7,8,9]. The earliest studies using cell therapy for pain relief tested adrenal chromaffin cells from rat or bovine sources [10,11]. The grafts placed in the subarachnoid space functioned as cellular minipumps, secreting a cocktail of antinociceptive agents around the spinal cord. Our previous study indicated that intrathecal implantation of PC12 cells reduced cold allodynia in a neuropathic pain model in rat [12]. However, ethical concerns have prevented the use of xenogenic (animal) chromaffin cells for pain therapy in humans. Clinical trials using human adrenal chromaffin cells gave promising results in patients with intractable cancer pain [9,13], in correlation with met-enkephalin and norepinephrine released into the cerebrospinal fluid (CSF). However, lack of a homogeneous, expandable cell source to supply the antinociceptive agents remains as a major problem. Pheochromocytoma is catecholamine-secreting tumor that arises from chromaffin tissue within the adrenal medulla and extra-adrenal site. A previous study has reported that human pheochromocytoma cells synthesize and secrete met-enkephalin and norepinephrine [14]. Therefore, we hypothesized that primarily cultured human pheochromocytoma cells would be a promising candidate for cell therapy to alleviate the intractable pain in cancer patients.

Given the antigenicity of the diverse cell source, purification and immunoisolation of grafts is necessary before transplant [15,16]. In addition, immunosuppressive therapies pose considerable risk to recipients and are harmful for the transplanted cells. Microencapsulation using alginate hydrogel has been widely used to overcome immune rejection and prevent the need for immunosuppressive therapy [17,18,19]. Although there is a physical separation of the transplanted cells from the host, small molecules such as erythropoietin and fibroblast growth factor can easily pass through the alginate gel barrier [20,21]. In a previous study, we have shown that met-enkephalin and norepinephrine in the CSF significantly increased following intrathecal implantation of microencapsulated PC12 cells [11]. The aim of the current investigation was to determine whether intrathecally implanted microencapsulated human pheochromocytoma cells (micro-HPC) could reduce pain in the rat model of bone cancer.

## 2. Results and Discussion

### 2.1. Bone Destruction Evaluation by Radiograph

**Figure 1:**
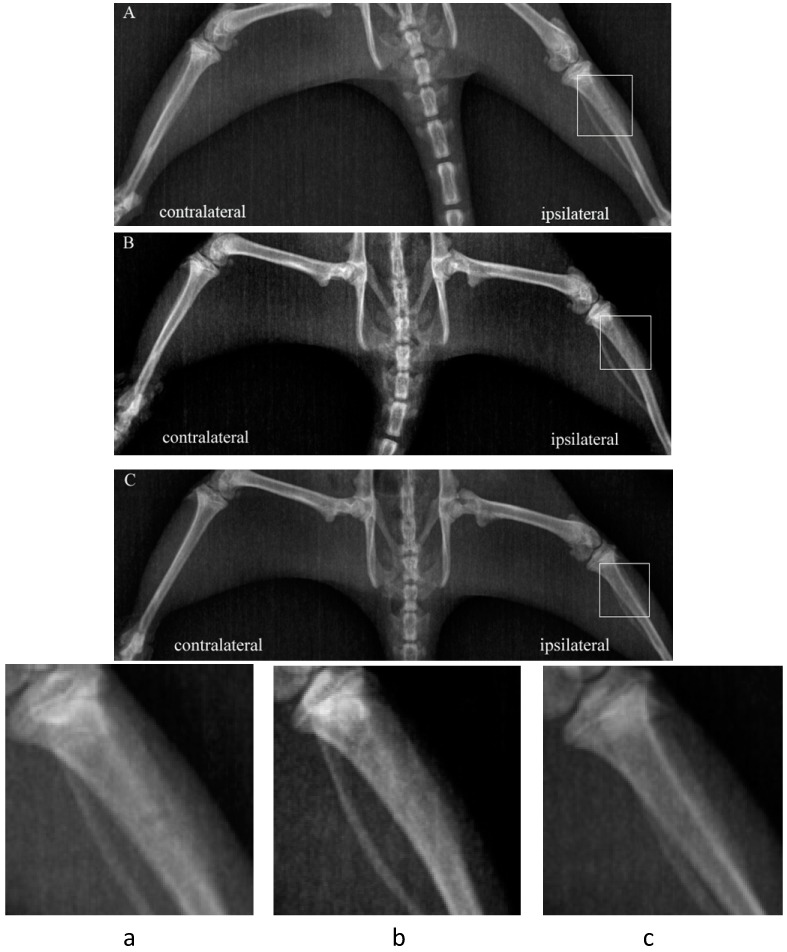
Radiological images of bone cancer-bearing tibiae of rat. (**A**) 7 days after implantation of walker 256 cells, obvious periosteal reaction was detected; (**B**) On day 21 after tumor implantation, significant cortical bone defect was observed in the cell-inoculated tibia bone; and (**C**) There were no radiological changes were observed in the ipsilateral tibia bone following culture media injection on day 21. (**a**–**c**) showing the proximal end of the ipsilateral tibia bones (**A**–**C**) with a higher magnification. No radiological changes were observed in the contralateral tibia bone. The white box indicates the injection site.

As shown in Figure 1, Walker 256 carcinoma causes progressive destruction of the tibia bone. Seven days after Walker 256 inoculation, the tibia bone showed obvious periosteal reaction in the proximal epiphysis (Figure 1A). Osteopenia and significant cortical bone defect was observed in the cell-inoculated tibia bone by day 21 (Figure 1B). No radiological changes were observed in the ipsilateral tibia bone following culture media injection (Figure 1C) or in the contralateral tibia bone (Figure 1A–C) up to 21 days after cell inoculation.

### 2.2. Time-Course of Mechanical Hyperalgesia Induced by Walker 256 Inoculation

**Figure 2:**
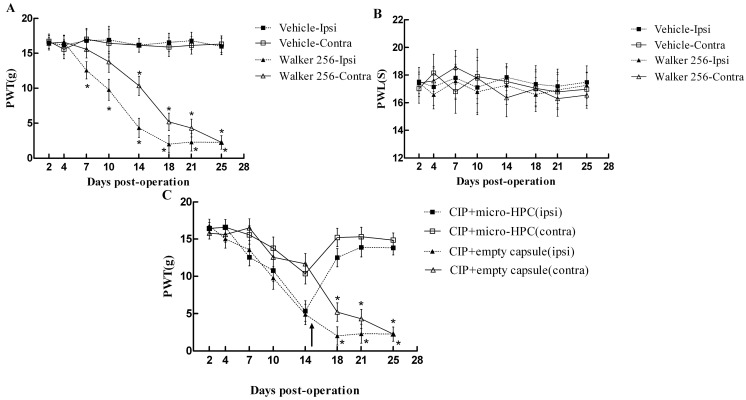
(**A**) The time course of the development of mechanical hyperalgesia after Walker 256 cell inoculation. Intra-tibial inoculations of Walker 256 produced a significant decrease of PWT (paw-withdrawal threshold) to von Frey filaments compared with the vehicle group (*p* < 0.05) on both hind paws. Mechanical allodynia occurred from day 7, peaked on day 18 and maintained up to the end of the experiment (day 25) on the ipsilateral side of bone cancer; (**B**) Rats injected with walker 256 cells showed no significant change in paw withdrawal latency to radiant heat stimulation on both hind paws throughout the experiment (p > 0.05); and (**C**) Micro-HPC (microencapsulated human pheochromocytoma cell) reduced established mechanical allodynia following intra-tibial inoculation of Walker 256. The PWTs on both hind paws were significantly increased in rats of cancer induced bone pain accepted micro-HPC compared with the empty capsules group (*p* < 0.05) at days 18, 21 and 25. Data are expressed as mean ± SD, *n* = 6 in each group. Significance indicated with * *p* < 0.05. PWL, paw-withdrawal latency; CIP, Cancer-induced bone pain.

Rats displayed significant reduced paw withdrawal threshold to the mechanical stimulus on the ipsilateral and contralateral side to the cell inoculation. Mechanical allodynia occurred from day 7, peaked on day 18 and maintained up to the end of the experiment (day 25) on the ipsilateral side of bone cancer, compared with the culture media (vehicle) injection group (*p* < 0.05) (Figure 2A). Mechanical allodynia was also detected in the contralateral hind paw from day 14 following cell inoculation and persisted throughout the experiment (*p* < 0.05, Figure 2A) No significant difference in the withdrawal threshold was observed in the both hind paws of rats receiving vehicle injection.

In agreement with previous reports, in the thermal hyperalgesia test, no significant reduction in threshold latency to thermal stimulus was detected in both vehicle and Walker 256 groups throughout the experiment (Figure 2B).

### 2.3. Secretion of Micro-HPC (Microencapsulated Human Pheochromocytoma Cell) in Vivo and in Vitro

It is well known that pheochromocytoma cells can secret large amounts of met-enkephalin and norepinephrine. Therefore, we firstly examined the concentration of met-enkephalin and norepinephrine in the culture media containing encapsulated human pheochromocytoma cells* in vitro*. ELISA (enzyme-linked immunosorbent assay) results revealed that the concentration of met-enkephalin and norepinephrine in the culture media containing human pheochromocytoma cells with or without microencapsulation were consistent for one week (Table 1). Furthermore, there was no significant difference in the concentration of met-enkephalin and norepinephrine between the two groups (*p* > 0.05). These data suggested that secretion was maintained in the microencapsulated human pheochromocytoma cells.

**Table 1:** The contents of met-enkephalin and norepinephrine in the cell culture media pre- and post-microencapsulation of human pheochromocytoma cell (5 × 10^4^ cells).

Time (Pre- and Post-Microencapsulation of HPC)	Met-Enkephalin (pg/µL)	Norepinephrine (ng/µL)
Pre-microencapsulation	3.14 ± 1.05	3.62 ± 0.61
Day2 (post-microencapsulation)	3.23 ± 0.79	3.59 ± 0.43
Day4 (post-microencapsulation)	3.09 ± 0.63	3.65 ± 0.95
Day6 (post-microencapsulation)	3.19 ± 0.48	3.54 ± 0.37

(*p* > 0.05) Results were obtained in adequate aliquots of pooled media from three individual wells recovered with medium change every two days. 100 microcapsules were studied (450~500 cells/capsule).

Next, we determined the concentration of met-enkephalin and norepinephrine in the cerebrospinal fluid (CSF) after intrathecal injection of the microencapsulated human pheochromocytoma cells in CIP model rats. ELISA results revealed a significant increase in the levels of met-enkephalin and norepinephrine in the CSF of rats that received micro-HPC injection compared to the empty capsule group (*p* < 0.05; Table 2).

**Table 2:** The concentration of met-enkephalin and norepinephrine in the CSF (cerebrospinal fluid).

Group	Met-Enkephalin (pg/µL)	Norepinephrine (ng/µL)
CIP + empty capsule	6.50 + 0.21	2.85 + 0.15
CIP + micro-HPC	8.93 + 0.65 *	5.54 + 0.73 *

Compared with the CIP + empty capsule group, * *p* < 0.05.

### 2.4. Intrathecal Injection of Micro-HPC Attenuated Mechanical Allodynia Induced by Walker 256 Inoculation

To investigate whether micro-HPC could reduce bone cancer pain, 100 microcapsules (450~500 cells/capsule) were administered through subarachnoid transplantation at day 15 following Walker 256 inoculation. The behavioral results showed that micro-HPC administration significantly increased the withdrawal threshold to the mechanical stimulus in the contralateral and ipsilateral paws compared with the Walker 256 group on days 18, 21 and 25 (*p* < 0.05, Figure 2C). Intrathecal injection of the empty microcapsule had no effect on the mechanical allodynia in the tumor-bearing rat. These results indicate that intrathecal injection of micro-HPC could reduce the mechanical allodynia induced by Walker 256 inoculation.

### 2.5. Antagonizing Opioid Signaling Reversed the Analgesic Effect of Micro-HPC on Mechanical Hyperalgesia

**Figure 3:**
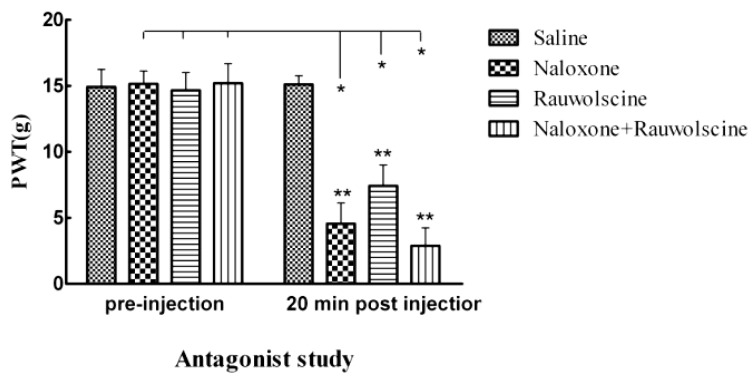
Effect of naloxone and rauwolscine on the degree of mechanical allodynia of bone cancer rats with micro-HPC. On day 20, all rats were first tested and separated randomly into four groups. The rats were then administered saline (10 μL, i.p.), naloxone (500 μg/kg, i.p.), rauwolscine (30 μg/10 μL, i.p.), and both naloxone and rauwolscine, and re-tested again 20 min later. Naloxone induced significant decreasing in PWT and rauwolscine partially changed the paw withdrawal behavior in rats having cancer pain with micro-HPC. The coadministration of naloxone and rauwolscine provided significant incremental reductions on PWT. The data are presented as mean ± SD. Intra-group comparisons (** *p* < 0.05), and comparisons between pre-injection and post injection of naloxone or/and rauwolscine (* *p* < 0.05) are made.

To determine the role of met-enkephalin and norepinephrine in the micro-HPC-induced analgesia, naloxone, an opioid antagonist, or rauwolscine, a α2-adrenergic antagonist, was administered 5 days after human pheochromocytoma cells transplantation and the mechanical withdrawal threshold was assessed after antagonist injection. The behavioral results showed that the mechanical withdrawal threshold was significantly reduced 20 min after naloxone (4.56 ± 1.49 *vs*. 15.12 ± 0.32 g, *p* < 0.05) and rauwolscine (7.42 ± 1.57 *vs*. 14.83 ± 0.93 g, *p* < 0.05) administration respectively compared with the pre-injection level (Figure 3). Moreover, coadministration with naloxone and rauwolscine provided significant incremental reductions on PWT (2.89 ± 1.39 *vs*. 15.09 ± 1.16 g, *p* < 0.05, Figure 3). These data indicate that the met-enkephalin and norepinephrine secreted by spinal implanted micro-HPC might mediate the anti-allodynia effect.

### 2.6. Discussion

The present study investigated the antiallodynia action of spinal implantation of microencapsulated human pheochromocytoma cells in the bone cancer model induced by intra-tibial inoculation of Walker 256. First, microencapsulated human pheochromocytoma cells were functional and secretion of met-enkephalin and norepinephrine persisted for at least 6 days* in vitro* and* in vivo*. Second, intrathecal implantation of microencapsulated human pheochromocytoma cells significantly inhibited reduced paw withdrawal threshold induced by bone cancer. Finally, the antiallodynic effect was reversed by administration of antagonist against met-enkephalin and/or norepinephrine. Our findings provided novel evidence that microencapsulated human pheochromocytoma cells might represent an alternative therapy for bone-cancer pain.

Animal models of bone cancer pain have been used to evaluate the efficacy and underlying mechanism of therapeutic interventions. Consistent with previous reports [22], our study showed that the animals demonstrated mechanical allodynia, which developed at 7 days, and peaked 21 days following inoculation of Walker 256 into the tibia, and was associated with progressive bone destruction. We used this model to evaluate the effect of human pheochromocytoma cells on chronic pain due to bone cancer.

Microencapsulation of cells as a useful technique for the delivery of bioactive molecules* in vivo* has been applied successfully in allogeneic or xenogeneic cell implants for several diseases, such as diabetes, liver failure and hypoparathyroidism [17,20,23]. The main advantage of microcapsules is to protect cells from a host’s immune system [24,25,26]. In addition, high mechanical resistance and biocompatibility of the elaborated microcapsules has also been observed in earlier reports [12,27,28]. Our previous study showed that intrathecal injection of APA-microcapsules of PC12 cells elicited no lympholeukocyte recruitment [12]. In the present study, we established for the first time the APA-microcapsule containing human pheochromocytoma cells. Furthermore, persistent higher concentrations of norepinephrine and met-enkephalin were detected in the culture of microencapsulated human pheochromocytoma cells or in the CSF after intrathecal implantation. These data provide reasonable evidence for subsequent functional studies of microencapsulated human pheochromocytoma cells.

Supplements with met-enkephalin and norepinephrine have been suggested as an alternative approach for pain treatment. In terminal cancer patients, implantation of adrenal medullary tissue has been shown to produce pain relief, which is associated with increased level of met-enkephalin and norepinephrine [9]. However, antinociception following chromaffin cell transplants produces controversial results in animal studies. For example, implantation of primary cultured human chromaffin cells has been observed in several chronic pain models [29,30], while bovine chromaffin cell grafts were shown to demonstrate no analgesic effect on acute or tonic pain in a rat formalin model [31,32]. It is suggested that the failure to produce antinociception might be due to inadequate amounts of grafted chromaffin cells, as well as low levels of met-enkephalin and catecholamines. In our experiment, ELISA assay demonstrated persistent high concentration of met-enkephalin and norepinephrine in the CSF after intrathecal implantation of microencapsulated human pheochromocytoma cells. In parallel with ELISA data, behavioral studies revealed significant reduced mechanical allodynia induced by bone cancer. These results confirmed that effective analgesia can be produced by intrathecal implantation of microencapsulated human pheochromocytoma cells.

It is well known that met-enkephalin produces analgesia via μ-opioid receptor and δ-opioid receptor [33,34,35], while norepinephrine potentiates the antinociceptive effect of μ-opioid receptor agonists by adrenergic α2 receptors [36]. To further determine whether the antinociception produced by microencapsulated human pheochromocytoma cells depends on μ-opioid receptor or α2 receptor, we evaluated the effect of a single injection of naloxone and/or rauwolscine on the analgesic effect. The inhibitory effect of naloxone or rauwolscine on the analgesia induced by human pheochromocytoma cells revealed that the antinociceptive effect is mediated by enhanced norepinephrine and met-enkephalin released from the transplanted cells. In addition, the total blocking effect of coadministration with naloxone and rauwolscine on the antinociception confirmed that met-enkephalin and norepinephrine may act synergistically to produce the analgesic following intrathecal implantation of human pheochromocytoma cells.

## 3. Experimental Section

### 3.1. Animals

A total of 126 female Sprague-Dawley (SD) rats, weighing between 200 and 250 g, were purchased from Medical Laboratory Animal Center of Sun Yat-sen University, Guangzhou, China. The rats were given 1-week acclimatization with free access to food and water under standardized housing conditions (12 h light-dark cycle, temperature of 22–24 °C, and relative humidity of 55% ± 5%). All animal experimental procedures were approved by the Sun Yat-sen University Animal Care and Use Committee and carried out in accordance with the guidelines of the National Institutes of Health on animal care and the ethical use.

### 3.2. Cells

Considering the characteristics of malignant tumor invasion and metastasis, pheochromocytomas without metastases were included in this study. And the tumors behaved in a benign fashion according to the Pheochromocytomas of the Adrenal gland Scaled Score (PASS) which is weighted for specific histologic features (PASS < 4) [37]. The pheochromocytoma tissues were obtained under aseptic condition and processed for primary culture as previously described [14]. Briefly, the pheochromocytoma tissue was minced into fragments measuring approximately 1 mm^3^. The minced tissue was placed in a sterile beaker and digested with 0.1% collagenase-I and 180 µg/mL deoxiribonuclease in Dulbecco’s Modified Eagle’s Medium/Ham’s F-12 (DMEM). The enzymatic digestion was performed at 37 °C under an atmosphere of 5% CO_2_ and 95% O_2_ for 10 min with constant stirring. At the end of the incubation period, the supernatant was collected by aspiration and transferred to a sterile tube. Cells were collected by centrifugation at 1000× *g* for 5 min at 4 °C and resuspended in 5 mL of red cell lysate and centrifuged at 1000× *g* for 5 min at 4 °C. The dispersed cells were maintained in DMEM (with 10% fetal bovine serum, 100 units/mL penicillin and 100 µg/mL streptomycin). The cells were incubated in the 5% CO_2_ cell incubator at 37 °C. Cell viability was determined by trypan blue staining.

### 3.3. Microencapsulation

Cell microencapsulation was performed using the coaxial gas flow bead generator (Nisco Engineering AG, Zurich, Switzerland) after a 48 h culture period. Cells were microencapsulated with alginate (NovaMatrix/FMC, Sandvika, Norway). Briefly, 10 × 10^9^ isolated cells were suspended in 10 mL of DMEM containing 1.5% sodium alginate. The suspension was extruded through a 26-gauge needle at a rate of 2 mL/h and dropped into 1.1% CaCl_2_ solution to form microcapsules of 200 to 250 µm diameter. After two washes in 0.9% NaCl, the microspheres were coated with 0.05% (*w*/*v*) poly-l-lysine (Sigma Chemical Co., St Louis, MO, USA) for 10 min and washed with 0.9% NaCl. Microcapsules were then exposed for 4 min to 0.15% sodium alginate which formed the outer layer of the membrane. The droplets were then washed twice in 0.9% NaCl and treated with 1 mmol/L sodium citrate (pH 7.4) for 2 min. After two washes in 0.9% NaCl, the encapsulated cells were distributed equally into six-well culture dishes with DMEM supplemented with 10% fetal bovine serum and maintained in the 5% CO_2_ cell incubator at 37 °C.

The microcapsules were implanted into the lumbar subarachnoid space. A posterior L5 laminectomy was performed, and a small incision was made in the dura. Approximately 100 microcapsules (450~500 cells/capsule) were implanted into the subarachnoid space via a 24-G Teflon catheter (Changzhou Helun Import & Export Co., Ltd., Changzhou, China) [38]. The catheter was maintained in place for 30 s after the injection, after which the incision was closed surgically. All surgical procedures were performed using aseptic conditions and anesthesia.

### 3.4. Bone Cancer Model and Radiology

Bone cancer was induced as previously described [39]. Briefly, under 0.4% sodium pentobarbital anesthesia (50 mg/kg, i.p.), a 1 cm rostro-caudal incision was made over the proximal half of the tibia. A 23-gauge needle was inserted into the intramedullary canal of the tibia, approximately 5 mm below the knee joint to create a cavity for injection of the cells, and a 10 µL volume of Walker 256 cells (approximately 2 × 10^5^ cells) or cell culture media was injected into the bone cavity using a 20 µL microinjection syringe. The cavity was sealed using bone wax and the wound closed with metal skin clips.

To assess bone destruction by cancer, tibia radiographs were performed in this study. Rats were placed on a clear plane plexiglass and exposed to an X-ray source under chloral hydrate anesthesia on days 7 and 21 after cancer cell inoculation. Using E-COM Digital Radiographer System (E-COM Technology Co., Ltd., Zhuhai, China), tibia radiographs were taken from contralateral and ipsilateral hind limbs of rats.

### 3.5. Mechanical Hyperalgesia Test

The paw-withdrawal threshold (PWT) to mechanical stimuli was measured using von Frey filaments with the up-down method, as previously described [40]. Briefly, rats were placed on an elevated wire grid and the plantar surface of the paw was stimulated with a series of ascending force von Frey monofilaments. Five minutes were allowed for habituation. Each test consisted of a 6–8 s application of the von Frey hairs with 5 min interval between stimuli. Quick withdrawal or licking of the paw in response to the stimulus was considered a positive response.

### 3.6. Thermal Hyperalgesia

Paw-withdrawal latency (PWL) to thermal stimulus was performed as previously described [41]. Rats were placed on the glass surface in a clear plastic chamber of the Paw Thermal Stimulator System (7370, UgoBasile, Comeria, Italy). After an adaptation period of 20 min, radiant heat was applied to the plantar surface of hind paw until the animal lifted its paw from the glass. The intensity of radiant heat was adjusted to elicit the response around 10 s in normal rats. A cut-off time of 15 s was imposed on the stimulus duration to prevent tissue damage. Mean PWL was established by averaging the latency of three tests with a 5-min interval between each test.

An observer blinded to the experiment design performed behavioral tests. Baseline threshold were obtained two days prior to the surgery. To evaluate the effects of the human pheochromocytoma cells on mechanical and thermal hyperalgesia induced by bone cancer, behavior tests were performed on days 2, 4, 7, 10, 14, 18, 21 and 25 after surgery. After the behavioral tests on day 25, the rats were killed with an overdose of sodium pentobarbital.

### 3.7. Secretion Study of Cells in Vitro and in Vivo

ELISA was used for measurement of met-enkephalin and norepinephrine in cerebrospinal fluid (CSF) or cell culture medium. To evaluate production of met-enkephalin and norepinephrine of human pheochromocytoma cells* in vitro*, 100 microcapsules (450~500 cells/capsule) per dish were cultured and media was collected every two days and stored at −80 °C until assays were performed. Met-enkephalin and norepinephrine were measured with ELISA assay kit (MyBioSource Inc., San Diego, CA, USA) according to the manufacturer’s protocol and the concentrations was calculated using a calibration curve.

After behavioral testing on day 25, animals were deeply anesthetized with sodium pentobarbital. An incision was made in the skin and muscle layers overlying the atlanto-occipital membrane. Then, CSF was extracted through the cisterna magna with a 30-gauge Hamilton needle. CSF samples were treated with 1× HALT Protease Inhibitor Cocktail (Pierce, Rockford, IL, USA) before ELISA performance to detect levels of met-enkephalin and norepinephrine.

### 3.8. Antagonist Study

In order to determine the mechanisms underlying the antinociceptive effect of micro-HPC in bone cancer-induced pain, we administered a non-selective opioid receptor antagonist naloxone (500 μg/kg, intraperitoneal injection), and a non-selective α2-adrenoceptor antagonist rauwolscine (30 μg/10 μL, i.p.). Mechanical hyperalgesia was evaluated at 20 min after antagonist administration in micro-HPC transplanted rats on day 20 after Walker 256 injection. The control group received the same volume of saline.

### 3.9. Statistical Analysis

All data were expressed as means ± standard deviations (SD). For analysis of ELISA data and antagonist study, differences between groups were compared by Student’s *t*-test or ANOVA (one-way analysis of variance) followed by Fisher’s PLSD post hoc analysis. For behavioral analysis, the data were compared with nonparametric test (Friedman and Bonferroni ANOVA for repeated measurements). The criterion for statistical significance was* p* < 0.05. Statistical tests were performed with SPSS 13.0 (SPSS Inc., Chicago, IL, USA).

## 4. Conclusions

Our present study demonstrated that microencapsulated human pheochromocytoma cells preserve the ability to secrete met-enkephalin and norepinephrine, and produce significant analgesia in a bone cancer pain model. Our data provide novel evidence that microencapsulated human pheochromocytoma cells might be a useful strategy for facilitation of clinical antinociception. However, the antinociceptive effect of transplantation of microencapsulated human pheochromocytoma cells need to be further investigated in other pain models.
